# Bioavailability of dietary polyphenols and plant bioactives in functional foods: insights and limitations from *in vitro* digestion models

**DOI:** 10.3389/fnut.2026.1900532

**Published:** 2026-08-18

**Authors:** Marwa Ezz El-Din Ibrahim, Utku Benan Usta, Seydi Yıkmış, Nazan Tokatlı Demirok, Marwah Nabeel Almaweed, Bodour Ibrahim Al Shik Mubarak

**Affiliations:** 1 Department of Food and Nutrition Sciences, College of Agricultural and Food Sciences, King Faisal University, Al-Ahsa, Saudi Arabia; 2 Nutrition and Dietetics, Faculty of Health Sciences, Tekirdag Namık Kemal University, Tekirdag, Türkiye; 3 Department of Food Technology, Tekirdag Namık Kemal University, Tekirdag, Türkiye

**Keywords:** bioaccessibility, bioavailability, Caco-2 permeability, colonic fermentation, dynamic digestion, INFOGEST

## Abstract

This review provides a comprehensive evaluation of how bioactive compounds within plant- and animal-derived food matrices interact with human physiology and metabolic pathways, with a particular focus on secondary metabolites synthesized or transformed via microbial pathways. The manuscript covers a diverse range of lipophilic and hydrophilic bioactive molecules, critically analysing these components from biological and technological perspectives. From a processing perspective, the mechanisms by which fermentation enhances antioxidant capacity by converting complex molecular structures into highly active aglycone forms are detailed. Concurrently, from an engineering perspective, the efficiency of nanoliposomes, solid lipid nanoparticles (SLNP), and biopolymeric carrier systems is assessed. Rather than claiming that encapsulation technologies can entirely overcome bioavailability limitations, this review critically assesses their potential to improve the stability, bioaccessibility and targeted release of sensitive components during gastrointestinal transit. Ultimately, by bridging microbial biotransformation with advanced delivery systems, this study offers a unified framework for optimizing the bioaccessibility of complex dietary compounds under simulated gastrointestinal conditions.

## Introduction

The health-promoting effectiveness of functional foods is closely related to how accessible their bioactives are and the way they are transformed during gastrointestinal transit. While plant-based polyphenols demonstrate significant biological activity *in vitro*, their actual systemic effects may be limited due to bioavailability, matrix interactions, and rapid intestinal metabolism (1). The molecular interactions between proteins and polyphenols primarily occur through weak, noncovalent interactions such as hydrogen bonds, hydrophobic interactions, and van der Waals forces. These interaction mechanisms can modify the structural integrity and biological functions of proteins in a modular, selective, and reversible manner. A review of recent literature reveals that studies on the formation dynamics of protein-polyphenol complexes and the stabilization of these non-covalent bonds have gained momentum, and this field has become critically important in the disciplines of food chemistry and biotechnology (2, 3). In natural matrices and industrial food systems, flavonoids tend to form complexes with proteins, as they are mobilized from the intracellular environment during the breakdown and processing of plant tissues. Today, increasing consumer awareness of preventive health and the resulting demand for functional foods are encouraging the inclusion of flavonoids in food formulations for enrichment. In this context, the interaction of flavonoids with protein-based carrier systems to maximize their bioavailability and functional properties has become central to modern food science research (4, 5). Protein-flavonoid interactions play a decisive role in protein conformation, potentially causing distortions, particularly secondary-structure elements. These structural changes directly affect the system’s overall emulsion stability and interfacial characteristics by reducing protein surface hydrophobicity and altering interfacial adsorption capacity. These physicochemical transformations resulting from the binding of polyphenolic compounds to protein chains pave the way for the reshaping of emulsifying properties in food matrices (6).

Although bioactive compounds are frequently cited for their health benefits, their oral administration presents significant challenges due to their low water solubility, high sensitivity to environmental factors during processing, and the demands of the gastrointestinal system, including gastric acidity and enzymatic degradation; this highlights the need to focus on bioavailability.

The primary objective of this review is to systematically examine the multidimensional effects of bioactive compounds present in plant- and animal-derived food matrices, particularly secondary metabolites synthesized or modified by microorganisms, on human physiology and metabolic processes. It aims to synthesize, in light of the literature, how fermentation processes increase antioxidant capacity by converting structurally complex compounds into more biologically active aglycone forms, and to examine the bioavailability dynamics of these components. The aim is to evaluate the technological effectiveness of nanoliposomes, solid lipid nanoparticles (SLNP), and biopolymeric carrier systems in overcoming the low stability and weak tissue-targeting capacity of sensitive bioactive compounds such as carotenoids and polyphenols (7, 8). This study demonstrates how these technologies optimize bioavailability by protecting the components from acidic and enzymatic degradation during gastrointestinal transit. The aim is to provide a comprehensive synthesis of current evidence on the international INFOGEST protocol (static, semi-dynamic, and dynamic models) simulating the digestion of food components, colonic fermentation approaches, and cell-based absorption models such as Caco-2. The aim is to contribute to the theory and methodology of the evaluation of the bioaccessibility of bioactive compounds in the design of functional foods. Furthermore, this critical review examines the modulation of polyphenol release, transformation and stability by plant-based food matrices (such as fermented legumes and plant products) under simulated upper gastrointestinal and Caco-2 cell-based digestion models.

## Materials and methods

This manuscript is structured as a narrative and critical review that synthesizes the complex interplay among food matrices, microbial biotransformation, and advanced delivery systems (9–11). To ensure scientific rigor, objectivity, and transparency in the literature selection process, a comprehensive search was conducted across major biomedical and scientific databases, including PubMed/MEDLINE, Scopus, and Web of Science (Core Collection).

The literature search was initially conducted from October 2025 to January 2026 and updated continuously until April 2026 to capture the most recent advancements in the field. The search strategy utilized combinations of MeSH terms, titles, abstracts, and keywords with Boolean operators (AND, OR) as follows: (“polyphenol*” OR “phenolic compound*” OR “flavonoid*” OR “bioactive compound*” OR “secondary metabolite*”) AND (“bioaccessibility” OR “bioavailability” OR “*in vitro* digestion” OR “INFOGEST “). To maintain a high degree of methodological quality, specific inclusion and exclusion parameters were applied during the narrative synthesis.

**Figure 1:**
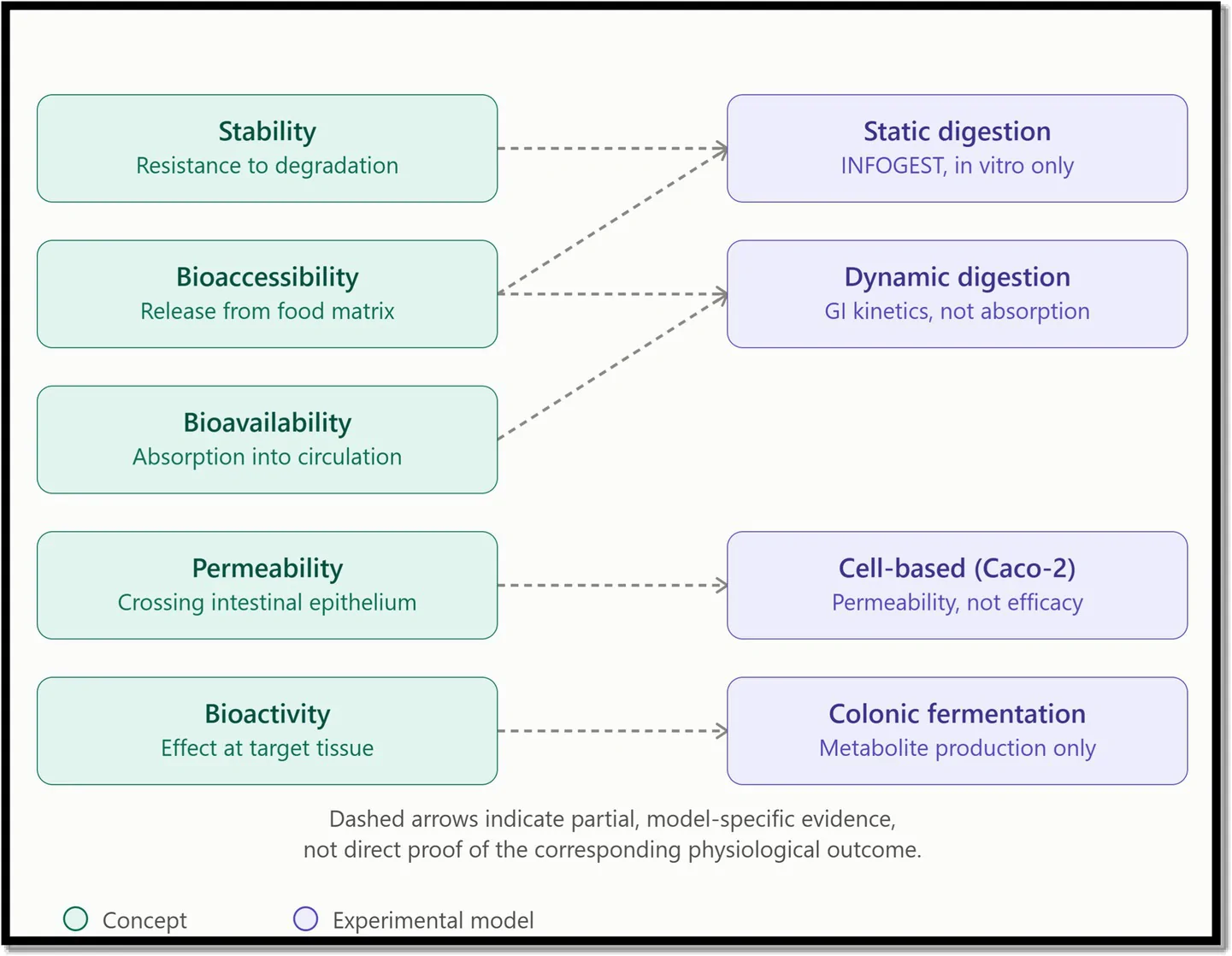
Conceptual relationships among bioaccessibility, bioavailability, permeability, stability, and bioactivity, mapped to the experimental model category most commonly used to approximate each construct. Arrows indicate partial, model-specific evidence rather than direct proof of the corresponding physiological outcome.

Given the rapidly evolving nature of the subject, studies from the last 10–15 years were prioritized; fundamental/classical studies deemed necessary were also evaluated for completeness. The retrieved literature was transferred to the reference management software, and the retrieved literature was reviewed to identify sources most relevant to the review focus. Priority was given to peer-reviewed articles (original research and qualified reviews) that directly reported bioavailability/bioaccessibility, included an *in vitro* digestion protocol, and/or presented biological activity outputs with post-digestion bioactive transformations. Sources with limited relevance to this scope were not considered further. From the included studies, the food matrix, bioactive class, model type used (static/dynamic/*colonic phase*/cell-based), digestion conditions (enzyme–pH–time–bile salts, etc.), measured outputs (bioavailability percentage, solubility/stability, phenolic profile change, antioxidant capacity, permeability, etc.) and the key findings were grouped together thematically and presented using a qualitative narrative approach. As shown in Figure 1, the relationships between these constructs are illustrated, with each mapped to the most common experimental model category for approximation. It should be noted that no single *in vitro* model fully captures a given construct in isolation, and a comprehensive assessment of bioaccessibility, bioavailability, and bioactivity typically requires complementary approaches.

### Functional food ingredients

The bioactive compounds in functional foods, particularly secondary metabolites such as flavonoids, carotenoids, and phenolic acids, form the basis for the development of next-generation functional foods and therapeutic (medical) products of critical industrial importance. These compounds are generally referred to as phytochemicals in botanical literature. Phytochemicals are chemical compounds produced by plants through primary and secondary metabolic pathways and exhibit significant biological activities. Sources include fruits (e.g., strawberries, pomegranates, and avocados), vegetables, nuts, seeds, legumes, and whole grains. In addition, seafood, olive oil, fermented foods, herbs and spices such as ginger, and beverages such as coffee, green tea, and black tea also contain these beneficial compounds (12).

### Plant-based functional foods

For carotenoids in plant matrices to become bioavailable, they need to be added to mixed micelles during the intestinal digestion phase. *In vitro* digestion models such as INFOGEST demonstrate that lipophilic bioactive release leads to significant matrix degradation, which facilitates cellular uptake in Caco-2 models (13).

However, preformed vitamin A (retinol and its derivatives) is concentrated in animal-derived foods such as dairy products, eggs, fish, and organ meats. Although the pharmacological activities of carotenoids through their antioxidant, anti-inflammatory, and anti-carcinogenic potentials are widely discussed in the literature, research on the clinical validation and therapeutic certainty of these effects is still ongoing (14). This synergistic effect is summarized in Table 1.

**Table 1:** Classification and functional characterization of bioactive components.

Bioactive classification	Featured compounds and key functions	Basic dietary sources	Potential biological activity observed in cellular/animal models	Daily average intake (mg/day)	Preclinical experimental models & doses (*in vitro*/animal)	Level of evidence/primary model	Reference
Polyphenols (flavonoids)	Quercetin, anthocyanins, catechin derivatives, kaempferol.	Berries, apples, onions, green tea, cocoa, and citrus fruits. *EGCG (Green tea catechin)*	Cardiovascular protection, anti-inflammatory response, high antioxidant capacity, and vascular regulation.	300–600	800–4,000 mg/day (in pure or green tea extracts:10–360 mg/day)	Human (*in vivo*) RCTs & Animal models	(63–65)
Phenolic acids	Caffeic, ferulic, and gallic acid derivatives	Coffee, whole grain products, exotic fruits, spices, and extra virgin olive oil.	*In vitro* cytotoxic properties/potential metabolic modulating capacity; potential neuroprotective properties; antiviral; and antimicrobial properties	200–500	*In vitro*: 1–100 μM (Cellular cytotoxicity & antioxidant assays)Animal: 10–100 mg/kg/day (Rodent metabolic/inflammatory models)	Epidemiological and microbiota studies	(66)
Stilbenes	Resveratrol, piceatannol	Grapes, red wine, peanuts, berries	*In vitro* antioxidant and anti-inflammatory activity; bioaccessibility shown to improve with emulsion-based encapsulation	5–10	*In vitro*: Pickering emulsion digestion assays; Animal: 20–200 mg/kg/day	*In vitro* digestion models, animal studies	(31, 67, 68)

EGCG, epigallocatechin gallate; mg/day: milligram/day; g/day: gram/day.

Data obtained from animal and *in vitro* modeling, and cellular concentrations, should not be directly correlated with systemic exposure in human physiology and therefore with actual bioavailability. The preliminary and *in vitro* findings presented in this review should not be interpreted as direct clinical evidence or confirmed therapeutic efficacy.

### Milk-based functional foods

#### Probiotics

As many probiotic strains originate from food, what we eat directly affects their ability to colonise the gut and modulate polyphenol biotransformation (15). When polyphenol bioavailability is examined, probiotic strains—particularly lactic acid bacteria—are able to convert complex polyphenol glycoside molecules into bioavailable aglycones during digestion via enzymatic deglycosylation through the *β*-glucosidase pathway (16, 17).

In order to evaluate this probiotic-mediated deglycosylation step under physiologically relevant conditions, standardised digestion models are required. In this context, the current revisions to the INFOGEST static *in vitro* digestion protocol that incorporate brush border enzymes into simulations offer a more reliable way of capturing the sequential contribution of microbial and epithelial *β*-glucosidase activity to the release of polyphenol aglycones (18).

### Carrier and matrix systems that enhance bioaccessibility

#### Antinutritional compounds affecting polyphenol bioaccessibility

Non-nutritive bioactive compounds such as phytates and tannins found in legume matrices significantly affect polyphenol stability and bioavailability during *in vitro* gastrointestinal transit due to digestive enzymes. Legumes contain various components, referred to in the literature as “non-nutritive compounds” (NNCs), that can limit the bioaccessibility of macronutrients (protein and carbohydrates). Some of these substances, which can chelate minerals such as calcium, iron, copper, and zinc, may exhibit physiologically toxic effects or negatively affect the sensory quality of food. For example, tannins present in the structure can cause a characteristic bitterness and undesirable taste profiles in the product (19, 20). The antinutritional components (ANC) found in legumes include enzyme inhibitors such as trypsin and amylase inhibitors, lectins, tannins, and phytic acid (myo-inositol hexakisphosphate/IP6). In addition to oxalates, phenolic compounds, and saponins, *α*-galactooligosaccharides (*α*-GOS) such as raffinose, stachyose, and verbascose are also important members of this group (21). Together, these antinutritional components primarily modulate polyphenol bioaccessibility by affecting enzyme–substrate interactions and matrix binding during digestion, rather than acting directly on polyphenols. This synergistic effect is summarized in Table 2.

**Table 2:** *In vitro* bioactive profile of secondary metabolites from legume sources.

Secondary metabolite	Structural features	Adverse effects on the organism	Bioactive and beneficial properties	References
Protease inhibitors	They are protein structures that inhibit enzymes involved in digestion.	They reduce the rate of protein digestion.	It has been shown to exhibit *in vitro* immunomodulatory and anti-inflammatory activity, alongside antiproliferative effects reported in cell culture models.	(69, 70)
Lectins (phytohemagglutinin)	Proteins that irreversibly bind to carbohydrate molecules.	It restricts nutrient absorption; it can adhere to organs and blood vessels, leading to autoimmune disorders.	Its effects vary; some lectins have been associated with cytotoxic or antiproliferative activity *in vitro*, alongside enzyme inhibitory effects.	(71, 72)
Phytates (phytic acid)	They are negatively charged compounds that hold minerals such as zinc, iron, and calcium.	It makes it difficult for the body to absorb minerals.	It exhibits *in vitro* antioxidant activity, with preclinical evidence suggesting potential relevance to cardiovascular and metabolic pathways, alongside *in vitro* cytotoxic properties.	(21, 73, 74)
Phenolic compounds	Aromatic ring structures containing one or more hydroxyl groups.	It reduces the bioaccessibility of amino acids and the digestibility of proteins.	*in vitro* cytotoxic properties and Potential metabolic modulating capacity.	(75–77)
Saponins	They are molecules that contain a sugar chain linked to an aglycone via a glycosidic bond at the C-3 position.	It can cause hemolysis (the breakdown of blood cells), hinder nutrient absorption, and damage the sperm membrane.	It has been reported to exhibit *in vitro* immunomodulatory, hypocholesterolemic, hypoglycemic, and anti-inflammatory activity.	(21, 78, 79)
*α*-Galactooligosaccharides	They are low-molecular-weight sugars containing special bonds (*α*1 → 6) between galactose units.	It causes gas, cramps, and diarrhea.	It exhibits prebiotic properties, supporting the growth of beneficial bacteria such as Bifidobacterium and Lactobacillus.	(21, 80, 81)

**Figure 2:**
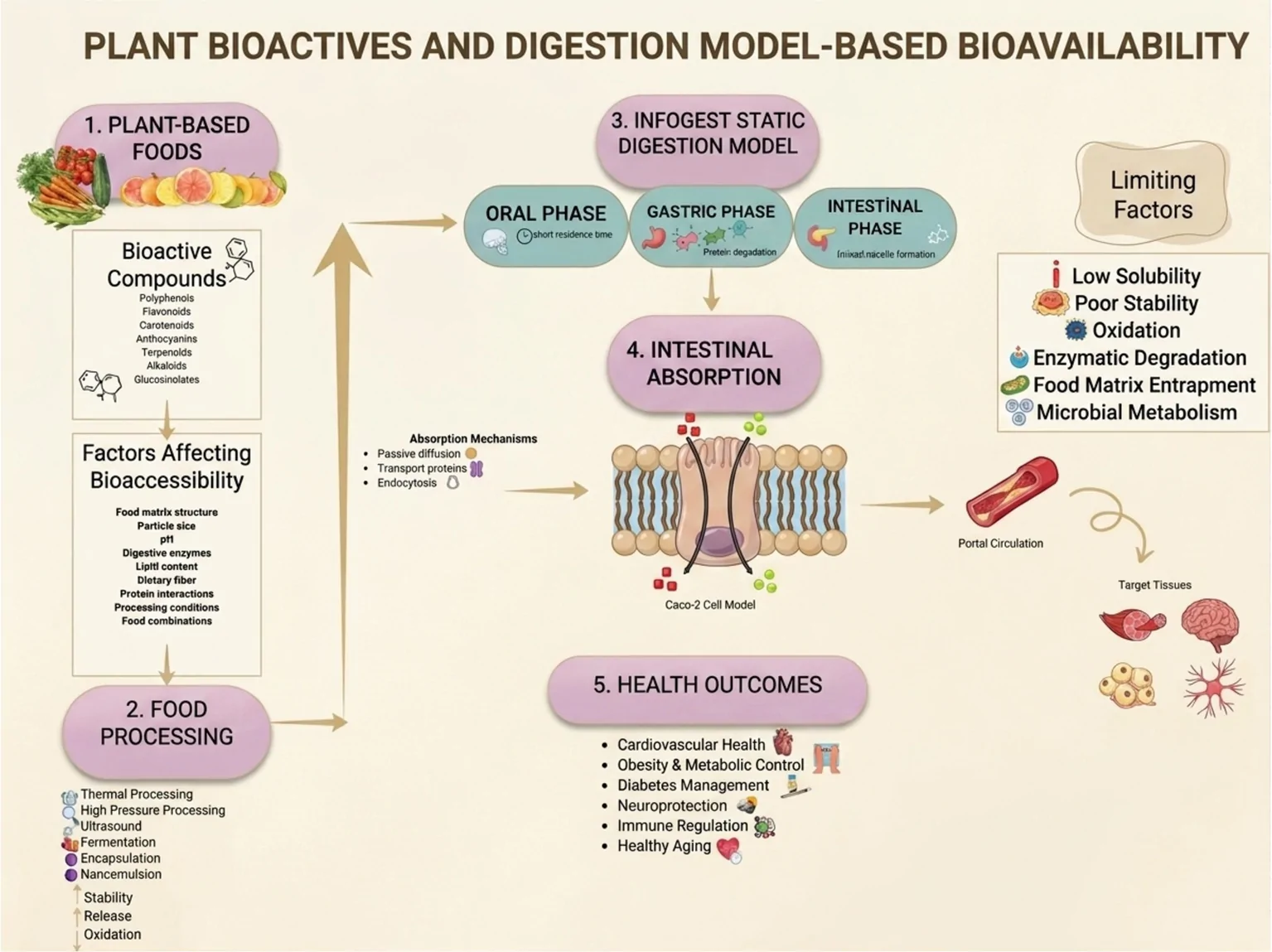
Schematic representation of the biotransformation processes during food fermentation and their physiological benefits on the human digestive tract and metabolic homeostasis (58–60).

The health-promoting potential of fermented foods largely depends on the synthesis of ‘*de novo*’ (new) bioactive compounds that are not initially present in the raw material matrix during the fermentation process. Scientific data indicate that the regulatory effects of fermented products on metabolic homeostasis are statistically more significant and stronger than those of unprocessed raw materials. This is due to the breakdown of complex polymers into smaller molecules with higher bioaccessibility, the synergistic effect of changes in factors that alter nutrient absorption and certain postbiotic compounds released as a result of microbial metabolism (22). This synergistic effect is summarized in Figure 2.

### Functional use of fermented vegetables

In addition to their unique organoleptic profiles, fermented fruit, vegetables and soy-based foods have a wide range of bioactive properties and are important in the global culture of nutrition. During fruit fermentation, degradation occurring via bacterial biotransformation has been shown to increase the recovery of free phenolic acids in the digested fraction and enhance post-digestive antioxidant activity *in vitro* intestinal cell modeling (23).

The functional properties of fruit and vegetable-based fermented foods are based on the metabolic activities of a complex microbial consortium consisting primarily of lactic acid bacteria such as *Lactobacillus plantarum*, *Pediococcus acidilactici*, and *Leuconostoc mesenteroides*, as well as yeasts such as *Saccharomyces cerevisiae* (24). During fermentation, the increased microbial activity raises the concentration of polyphenolic compounds and flavonoids in the matrix, thereby boosting its *in vitro* antioxidant capacity (25, 26).

Kimchi, one of the fundamental gastronomic components of Korean cuisine, is categorized as a ‘superfood’ in functional food literature due to its complex fermentation dynamics. Throughout the fermentation process, the population density of *lactic acid bacteria (LAB)* such as *Lactobacillus brevis*, *Limosilactobacillus fermentum*, *Lactiplantibacillus plantarum*, *Leuconostoc mesenteroides*, and *Weissella confusa*, and the concentration of secondary metabolites, show a logarithmic increase. During fermentation, lactic acid bacteria produce *β*-glucosidase enzymes that break down polyphenol glycosides into smaller, more lipophilic aglycones. This structural change is believed to promote passive diffusion across the intestinal epithelium. Additionally, kimchi-associated LAB strains have been shown to protect Caco-2 tight junctions against oxidative and inflammatory stress, thereby reducing paracellular permeability loss. Nevertheless, there is still a lack of direct evidence demonstrating aglycone transport under standardised INFOGEST-Caco-2 conditions (27–29).

### Lipid-rich functional foods and emulsified systems

#### Emulsions and structured lipid systems

**Figure 3:**
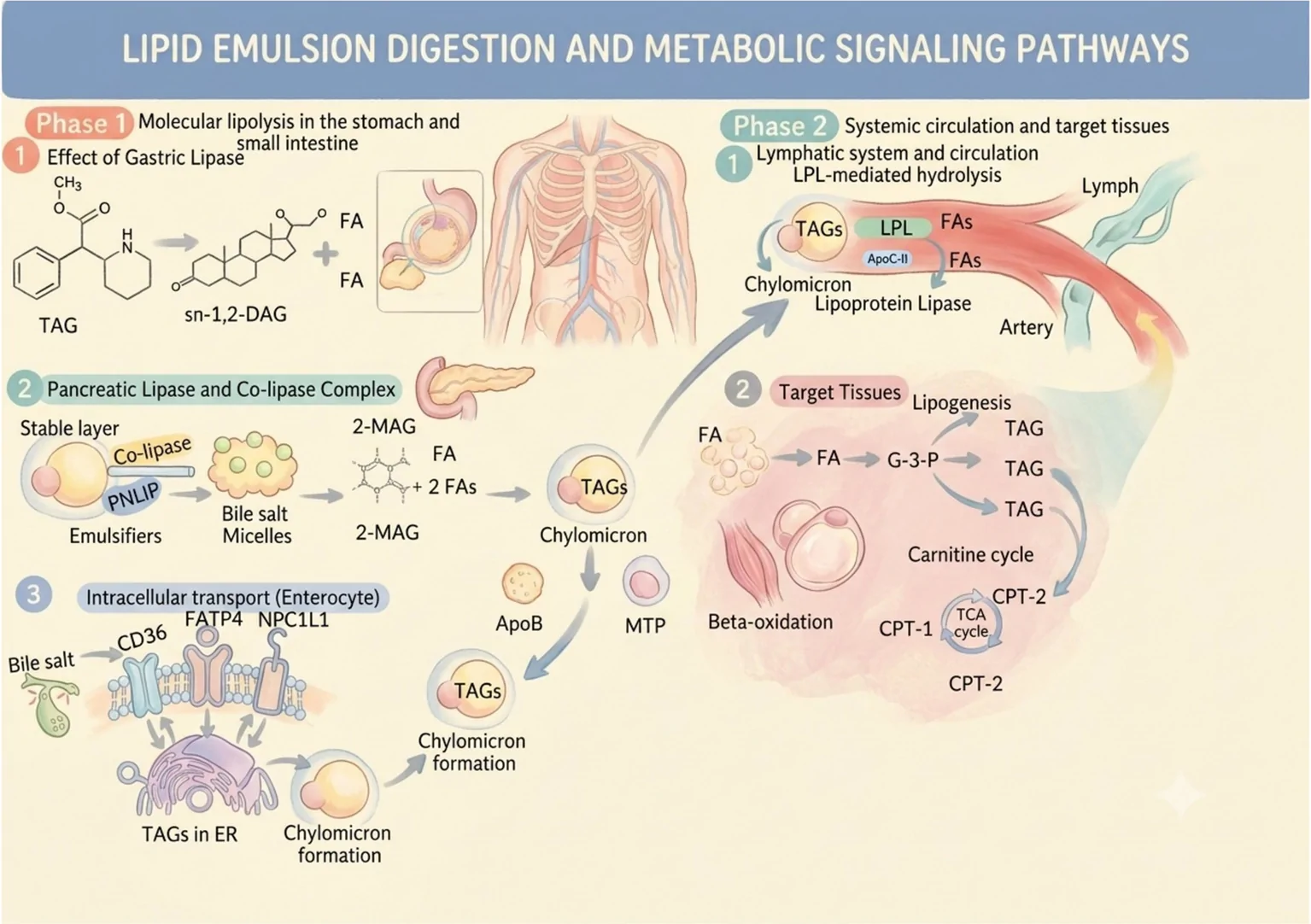
Structural classification and delivery mechanisms of lipid-based colloidal systems (O/W and W/O emulsions) for the stabilization and bioaccessibility enhancement of lipophilic bioactives (61, 62).

Emulsification and stabilisation technologies are also utilised to incorporate lipophilic polyphenols, for example curcumin, resveratrol and quercetin, into food systems (30). Encapsulating these compounds within emulsion-based delivery systems not only stabilises them against environmental and digestive degradation, but also significantly enhances their oral bioaccessibility and absorption (31). The detailed molecular mechanisms of luminal lipolysis, enterocyte transport, and systemic metabolic pathways of lipid emulsions are depicted in Figure 3.

Emulsions are defined as colloidal systems consisting of at least two immiscible liquid phases that are thermodynamically unstable. In these systems, one phase is dispersed within the other at the micron- or nanoscale using surfactants (surface-active agents) and advanced homogenization techniques. Systematically, the liquid dispersed in the form of particles is called the ‘dispersed (inner) phase, while the environment surrounding these particles is called the ‘continuous (outer) phase (32). The primary liquid components in food-grade emulsion formulations are the oil and water phases. Based on the distribution of geometry of these phases, two fundamental systems are defined. Table 3 summarizes these oil-in-water and water-in-oil emulsion systems and their relevance to bioactive delivery. The bioavailability of encapsulated lipophilic bioactive substances is directly influenced by their O/W and W/O configurations; exposure to gastric/intestinal lipases and incorporation into mixed micelles during simulated digestion are important factors.

**Table 3:** ‘Oil-in-water’ (O/W) emulsions and ‘water-in-oil’ (W/O) emulsions, where water droplets are dispersed within the oil phase (82, 83).

Feature	Oil-in-water (O/W)	Oil in water (W/O)
Distributed phase	Oil droplets	Water droplets
Continuous phase	Water	Oil
Primary function	Transport of hydrophobic components	Dispersion of water-based components in oil
Applications	Lipophilic polyphenols (e.g., curcumin, resveratrol), vitamins, essential fatty acids	Margarine, low-fat spreadable products

#### Edible oils and oil-based functional formulations

Current studies in the literature have focused on integrating essential oils such as rosemary, palmarosa, patchouli, eucalyptus, artemisia, thyme, and oregano into nanoemulsion systems. These studies primarily focus on the characterization processes and physicochemical stability of these systems. In simulated gastrointestinal digestion models, the bioaccessibility of the encapsulated compounds is significantly influenced by lipase-mediated hydrolysis and micelle formation, both of which depend on nanoemulsion droplet size and interfacial composition. Incorporating these nanoemulsions into food matrices therefore has direct implications for their bioaccessibility and, ultimately, bioavailability (33, 34).

### Functional beverages and infusions

INFOGEST-based models are used to evaluate the significant degradation of green tea catechins in the stomach and intestines (35). Coffee is a major dietary provider of chlorogenic acids and other phenolic compounds, released during infusion. Studies have demonstrated that coffee is one of the dietary elements that contributes most to total daily polyphenol ingestion. In this respect, coffee has been connected with lower levels of oxidative stress in various observational studies (36).

Bioactive polyphenols generally exhibit stability under gastric conditions but undergo significant structural degradation in the alkaline intestinal phase (37). Furthermore, their interactions with components such as proteins and carbohydrates significantly affect their solubility and ultimate bioaccessibility (37–39).

Several clinical studies have reported that cocoa-derived polyphenolic compounds may reduce circulating markers of systemic inflammation (40) These bioactive profiles are summarized in Table 4.

**Table 4:** Bioactive components and reported preclinical/biological activities of functional beverages.

Beverage type	Featured compounds	Daily average intake (mg/day)	Preclinical experimental models & doses (*in vitro*/animal)	Evidence level	References
Green tea	Catechin derivatives (especially EGCG), caffeine, quinic acid, phenylacetic acid, l-glutamine, and ascorbic acid.	90–300 (habitual); up to 866 (high consumers)	*In vitro*: 1–100 μM (cell-based mechanistic assays); Animal: 50–100 mg/kg/day (oral, mouse)	Human (observational, dietary intake)/preclinical (*in vitro*, animal)	(84–86)
Coffee	Alkaloids (caffeine), chlorogenic acids, diterpenes (cafestol, kahweol), and polymeric melanoidins.	500–1,000	*In vitro*: 25–113 μM (antioxidant/ROS assays); Animal: 10–100 mg/kg/day (oral, rat)	Human (dietary intake data, EFSA)/preclinical (*in vitro*, animal)	(87, 88)

EGCG, epigallocatechin gallate.

Plant-based milk products derived from kidney beans (*Phaseolus vulgaris*) have been shown to retain antioxidant activity and measurable *in vitro* phenolic bioavailability; thus, they have potential as functional beverage alternatives (41).

### Encapsulated functional foods and delivery-optimized products

The peels and seeds that constitute the primary by-products of the fruit processing industry possess high-value-added components, granting them significant application potential across the food, cosmetic, and pharmaceutical (drug) sectors. The extracts obtained from these waste streams are considered critical raw material sources for circular economy models within the “zero waste” principle, thanks to their rich content of bioactive substances (42). These technological limitations and bioactive losses can be overcome by microencapsulation techniques that isolate sensitive components from adverse environmental conditions and form a protective matrix. This method increases the resistance of bioactive substances to external factors, thereby stabilizing their chemical stability and enabling controlled release mechanisms (43, 44).

### Liposomal and biopolymeric delivery formats

Polyphenolic compounds have become a major focus of encapsulation research due to their antioxidant properties and their susceptibility to degradation during digestion and storage. However, their low stability and limited tissue-targeting capacity significantly limit their bioaccessibility and functional efficacy. Nanoliposomes, developed to overcome these limitations, offer an effective encapsulation strategy for protecting active components, thanks to their unique architectures, high biocompatibility, and controllable release mechanisms. Nanoliposomal systems optimize both chemical stability and delivery efficiency to the target region by protecting sensitive components from environmental stressors (45). Encapsulation technology is recognized as a fundamental strategy for the systematic design of engineering solutions for the agriculture-food industry, particularly in areas such as functional food formulation, food processing, and product innovation. This technique is based on the principle of encapsulating a core material exhibiting bioactive properties operationally within an inert matrix (wall material) with protective qualities, thereby isolating it from the external environment (46). Microencapsulation technology has found a wide range of industrial applications due to its ability to protect sensitive and unstable bioactive components from adverse environmental factors encountered during production, processing, and storage, such as moisture, photooxidation (light), and thermal fluctuations. Beyond preserving the chemical stability of components, this technology enables the integration of innovative features, such as controlled release and targeted functionality, into designed food products (47). A comparison of these microencapsulation and delivery systems is summarized in Table 5.

**Table 5:** Comparison of microencapsulation and delivery systems for polyphenol stabilization.

Carrier system	Objective	Expected bioaccessibility effect	Main limitations	References
Spray drying	To stabilize heat-sensitive (thermolabile) components and extracts by converting them into micronized powder form.	It provides protection during gastrointestinal transit by increasing the resistance of bioactive substances to external factors (oxygen, light).	The selection of wall material used during processing and the thermal sensitivity of the core material.	(89–91)
*SLNP*	To enable the transport of hydrophobic (water-insoluble) bioactive components and provide precise controlled release kinetics.	Provides high physicochemical stability, enabling controlled release of bioactives within the body for a longer period.	Tendency to crystallize and the associated risk of the loaded material leaching out over time.	(92)
Biopolymeric nanoparticles (PLA and Chitosan)	Ensuring high bioactivity and structural stabilization in nanomedical applications through bio-compatible matrices.	Prevents enzymatic degradation and provides controlled/extended release thanks to its polymeric matrix structure.	The biodegradation rate of the polymer and the complexity of the production methodology (nanoparticle design).	(93)

SLNP, solid lipid nanoparticles; PLA, polylactic acid.

### Bioaccessibility trends

To date, human-focused *in vivo* studies examining the effects of gut microbiota (GMB) on glucosinolate (GSL) metabolism have been limited (48). The modulatory effects of red beet and red cabbage microgreens on GMB were investigated in a healthy middle-aged and elderly population. The analysis results show that dietary intake of these microgreens did not significantly alter gut microbial community structure. Bouranis and colleagues emphasized that the biotransformation of glucosinolate (GSL) metabolites is primarily carried out by bacteria belonging to the *Bifidobacterium* and *Roseburia* genera. In this study, which analyzed sulforaphane (SFN) levels in plasma and urine samples and microbiota sequencing data in stool samples from a total of 55 participants, it was observed that a diet rich in carbohydrates, fiber, and micronutrients supports a gut microbiome (GMB) composition that promotes glucoraphanin (GR) hydrolysis (49). The inadequacy of *in vivo* studies has led researchers to explore various *in vitro* models; the findings from these studies have provided an important theoretical basis and methodological guidance for future clinical studies. Sulforaphane (SFN), isolated from broccoli, was subjected to simulated *in vitro* gastrointestinal digestion processes. Following confirmation that SFN stability was maintained during digestion, *in vitro* fermentation analyses revealed that SFN levels decreased significantly during the first 6 h and then remained stable. Microbiota analyses performed at 24 h of incubation showed that the gut microbiota (GMB) composition improved in the SFN-treated group compared with the control group. This improvement was recorded as an increase in the Firmicutes phylum, one of the most abundant beneficial taxa, and a marked decrease in *Proteobacteria*, which is associated with intestinal dysbiosis (imbalance) (50). Olive tree (*Olea europaea* L.) derivatives, which form the cornerstone of the Mediterranean diet (MD), particularly extra-virgin olive oil (EVOO) and table olive consumption, have become indispensable indicators of a healthy lifestyle today. The biological activity and protective effects of olive-based products on human health stem from their unique and rich chemical composition (51). Olive oil (OO) is characterized by a unique composition consisting primarily of unsaturated fatty acid esters such as oleic, linoleic, and linolenic acids. However, beyond the lipophilic fraction of OL, another group of components has become the focus of scientific studies in recent years: olive oil polyphenols (OP). These compounds have gained strategic importance in biomedical research due to their high biological activity capacity (52). In addition, oleuropein (Oleu), a secoiridoid glycoside found in high concentrations in olive fruit and leaves but only in trace amounts in olive oil, is described in the literature as a critical bioactive compound. A large number of preclinical studies have reported the antioxidant and bio-protective properties of oleuropein that are important for human health (53). Although various scientific approaches have been proposed to investigate the metabolic processes of bioactive compounds derived from food sources, this field remains largely understudied. This situation is particularly evident for olive oil bioflavonoids (OOB); despite extensive bibliographic data from *in vitro*, *in vivo*, and clinical studies on their biological activities, research on their bioaccessibility and metabolic fate remains quite limited. To determine the metabolic profile of a compound, enzyme-based systems such as microsomes, superosomes, cytosol, and S-9 fraction, as well as cell-based models (primary hepatocytes, liver slices, and perfused liver) that evaluate intestinal permeability and hepatic metabolism are routinely used (54). Therefore, elucidating the bioaccessibility and metabolism of OOB requires comprehensive research and multidisciplinary experimental approaches to address uncertainties in the literature and obtain reliable data. In this context, the present study comprehensively evaluates *in vitro, in vivo,* and clinical research investigating the absorption, distribution, metabolism, and excretion (ADME) mechanisms of OOBs (55).

### *In vitro* digestive models

The digestion of macronutrients begins with hydrolysis, which occurs via substrate-enzyme interactions. The resulting hydrolysis products, such as amino acids, sugars, and fatty acids, are absorbed through the brush border of the intestinal epithelial cells and transferred into the systemic circulation. At this stage, molecular hydrophobicity is the key factor determining the transport mechanism: Hydrophilic components (sugars, short-chain fatty acids, etc.) are transported via the portal vein, while lipophilic molecules (curcumin, carotenoids, long-chain fatty acids, etc.) are packaged within chylomicrons and transferred via the lymphatic system. These transport pathways directly affect the bioaccessibility of bioactive components (56). Proteins are macromolecules that play a fundamental role in critical biological processes, including maintaining structural integrity, facilitating molecular transport, and catalyzing biochemical reactions. Consuming dietary proteins has beneficial effects on human health. Protein digestion, particularly of bioactive peptides and amino acids, is of strategic importance for regulating metabolic functions and maintaining homeostasis (57). To determine the fate and biological activity of dietary proteins in the gastrointestinal tract, it is critically important to systematically analyze their digestion profiles using *in vivo* or *in vitro* models. Various *in vitro* simulation strategies are used to analyze the digestibility capacity of plant-based proteins (PBP). Unlike relatively simple static models that provide basic data at the endpoints of the digestion process, semi-dynamic models more closely mimic the kinetic nature of in vivo gastric digestion and achieve higher accuracy. These advanced models integrate critical parameters, such as stepwise acidification, dynamic enzyme and fluid secretion, and gastric emptying rate, and present the hydrolysis kinetics and time-dependent structural changes of proteins from a more realistic perspective (8). A methodological comparison of these simulated digestion models and their physiological relevance is summarized in Table 6.

**Table 6:** Methodological comparison and physiological relevance of simulated digestion models.

Model type	Physiological relevance	Strengths	Limitations	Target compounds	Recommended outputs	Key references
Static *in vitro* models (e.g., INFOGEST 2.0)	*In vitro* oral, gastric, and intestinal phases are simulated under controlled conditions characterized by fixed pH values and predetermined enzyme ratios	Owing to its technical simplicity and economic viability, the developed method demonstrates exceptional reproducibility and high-throughput screening efficiency	This approach operates without accounting for temporal transit complexities, peristaltic contractions, physiological gastric emptying sequences, or kinetic pH adjustments.	The analytical framework characterizes the representative dietary constituents, comprising proteins, lipids, starches, and structurally resilient polyphenols.	Key post-digestive metrics: final micellar bioaccessibility (%) and macronutrient hydrolysis efficiency.	(7, 18)
Dynamic *in vitro* models (e.g., TIM-1, SHIME)	Physiological gastric emptying, real-time kinetic pH alterations, and peristaltic movements are accurately simulated within the system	Owing to its elevated physiological relevance, the apparatus closely recapitulates human mechanical digestion kinetics and gastrointestinal transit dynamics	Major bottlenecks of this platform include its intrinsic procedural complexity, low-throughput efficiency, and high operating costs relative to the amount of data generated, alongside the dependency on specialized infrastructure.	The analytical design integrates encapsulated bioactive delivery systems and probiotics into complex food matrices to assess gastrointestinal delivery efficiency	Kinetic parameters under monitoring: digestion kinetics, time-dependent release profiles, and population survival curves	(7)
Cellular coupled models (e.g., INFOGEST + Caco-2)	Following digestion, subsequent permeation through the intestinal epithelial barrier and internalization by cells are simulated	By integrating bioaccessibility matrices with cellular transport dynamics, this methodology addresses a fundamental transition in digestive physiology	The physiological fidelity of Caco-2 monocultures is restricted by the absence of a mucus matrix (goblet cells) and host first-pass metabolism, compounded by the potential cytotoxicity of digestive fluids on the cell monolayer	The post-digestive matrix was characterized by a high abundance of carotenoids, free amino acids, and small peptides, alongside bioaccessible trace minerals	“Parameters for barrier function and bioaccessibility: relative cellular uptake (%) and transepithelial electrical resistance (TEER)	(7, 94, 95)

INFOGEST: An international network of in vitro digestion protocols that standardizes digestion processes in a laboratory setting; TEER, transepithelial electrical resistance; TIM-1: TNO Gastrointestinal Model-1; SIMGI, simulator of the gastrointestinal tract; SHIME, simulator of the human intestinal microbial ecosystem.

Differences in research focus and conceptualization of physiological parameters have led to methodological heterogeneity among *in vitro* models, complicating the comparability and systematic analysis of experimental data. To address these limitations, an internationally standardized protocol, INFOGEST 1.0, was developed in 2014. This protocol was updated to INFOGEST 2.0 in 2019, enhancing the simulation of the gastrointestinal microenvironment by including the oral phase and adding gastric lipase. This standardization has consolidated data from different laboratories and provided a widely adopted framework for comparing bioaccessibility outputs across studies (9–11).

### Limitations and future perspectives

While *in vitro* digestive and cellular models are useful in understanding the mechanisms of action of bioactive compounds, there are methodological limitations in understanding their bioaccessibility in human physiology. Caco-2 monocultures do not provide a clear representation of the complex and dynamic structure of the human intestinal barrier, including immune cell interactions and intestinal motility. In addition, enzyme activities, pH processes, and bile salt compositions do not provide a complete representation. Therefore, it is crucial that researchers validate *in vitro* findings with human pharmacokinetic studies and well-designed clinical intervention trials to demonstrate levels of clinically proven efficacy. Furthermore, most *in vitro* systems lack information on ongoing metabolic processes in the host (hepatic biotransformation, first-pass effects), do not account for variability in gut microbiota composition among individuals, and cannot fully determine the effects of food matrix complexity on bioactive release and stability; all these issues hinder the acceptance of *in vitro* bioaccessibility.

## Conclusion

Going beyond traditional methods for evaluating functional foods, this approach has revealed the decisive roles of molecular interactions and bioaccessibility dynamics in health-promoting effects. Fermentation is not only a food preservation method, but also a biotechnological process in which ‘*de novo*’ bioactive compounds (SCFA, postbiotics) not found in the raw material are synthesized. In this process, complex polyphenols and glycosides are converted to their aglycone forms, which are more biologically active, thereby maximizing the matrix’s total antioxidant capacity and functional bioactive potential. The low stability and poor tissue targeting capacity of bioactive components (especially lipophilic carotenoids and sensitive polyphenols) are the biggest obstacles facing the functional food industry. Nanoliposomes, *SLNP*, and biopolymeric carriers have been shown to optimize bioaccessibility by protecting these sensitive components from enzymatic and pH-induced degradation during gastrointestinal transit. These technologies facilitate the controlled release of bioactive substances, improving their delivery to target tissues under simulated digestive conditions. The combination of different plant matrices creates a synergistic antioxidant capacity that is much stronger than the sum of the components’ effects. In addition, non-covalent bonds, such as protein-polyphenol interactions, modify the physicochemical properties of the food matrix, reshaping both emulsion stability and the digestibility profile. In terms of methodological standardisation, the INFOGEST protocol, which is used to simulate digestive processes, is one of the international standards that has provided a widely adopted framework for improving the comparability of bioaccessibility data across studies. The transition from laboratory to industrial scale in distribution systems is of great importance, but high production costs, low scaling efficiency, and potential long-term toxicity or biocompatibility issues with synthetic nanomaterials present several limitations. Furthermore, standardized safety assessments for nanoencapsulated functional foods are crucial. Future research should focus on developing functional products tailored to individual microbiota profiles based on these standardized models and on proving the systemic effects of these components through long-term clinical studies. The recovery of agricultural industrial waste (such as husks, seeds, etc.) as a source of bioactive compounds offers a sustainable strategy for combating nutritional deficiencies while also providing the functional food industry with low-cost, high-value-added raw materials. The success of the functional food industry hinges on a multidisciplinary approach that integrates the selection of the right microbial strains, optimized fermentation kinetics, and advanced encapsulation technologies.
